# The results of arthroscopic anterior stabilisation of the shoulder using the bioknotless anchor system

**DOI:** 10.1186/1758-2555-1-2

**Published:** 2009-01-19

**Authors:** Stephen J Cooke, Ian Starks, Vinod Kathuria

**Affiliations:** 1 Department of Trauma & Orthopaedics, Stafford General Hospital, Stafford, UK

## Abstract

**Background:**

Shoulder instability is a common condition, particularly affecting a young, active population. Open capsulolabral repair is effective in the majority of cases, however arthroscopic techniques, particularly using suture anchors, are being used with increasing success.

**Methods:**

15 patients with shoulder instability were operated on by a single surgeon (VK) using BioKnotless anchors (DePuy Mitek, Raynham, MA). The average length of follow-up was 21 months (17 to 31) with none lost to follow-up. Constant scores in both arms, patient satisfaction, activity levels and recurrence of instability was recorded.

**Results:**

80% of patients were satisfied with their surgery. 1 patient suffered a further dislocation and another had recurrent symptomatic instability. The average constant score returned to 84% of that measured in the opposite (unaffected) shoulder. There were no specific post-operative complications encountered.

**Conclusion:**

In terms of recurrence of symptoms, our results show success rates comparable to other methods of shoulder stabilisation. This technique is safe and surgeons familiar with shoulder arthroscopy will not encounter a steep learning curve. Shoulder function at approximately 2 years post repair was good or excellent in the majority of patients and it was observed that patient satisfaction was correlated more with return to usual activities than recurrence of symptoms.

## Introduction

**Figure 1:**
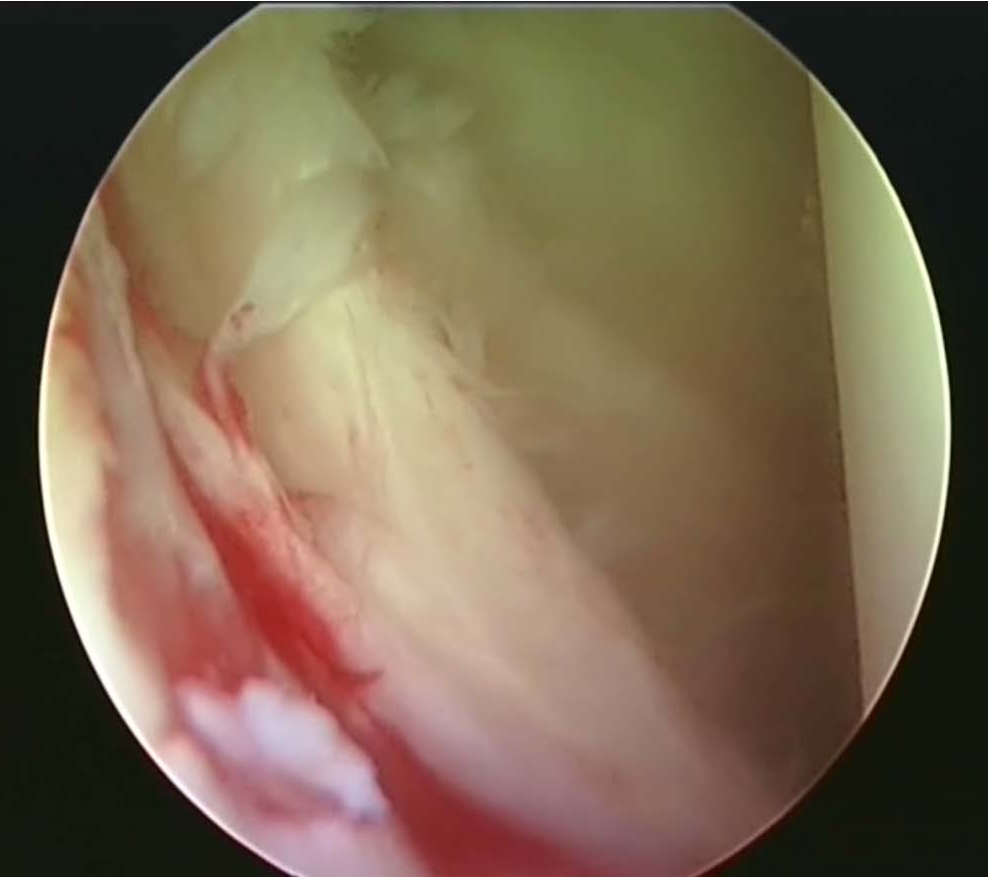
**Arthroscopic view of a Bankart lesion in a right shoulder**.

Stability of the shoulder relies on passive and dynamic constraints. Passive constraints include the bony anatomy, augmented by the glenoid labrum, capsule, ligaments and a negative intraarticular pressure. Dynamically, the rotator cuff and scapular musculature act to centralise the humeral head within the glenoid fossa throughout the normal range of motion of the shoulder joint. Instability can be defined as repeated dislocation or subluxation of the glenohumeral joint without extraordinary external force. Shoulder instability is a very common problem which predominantly affects a young, active population [1]. The most frequent pathology is the classic Bankart lesion (figure 1) following a traumatic anterior dislocation [2], but instability can also arise without trauma and can occur in any direction. Symptoms range from mild symptomatic instability to recurrent dislocation, with some patients able to voluntarily dislocate their own shoulders. Therefore shoulder instability represents a spectrum of conditions with different aetiologies and methods of management.

The evolution of minimally invasive surgery has led to the development of arthroscopic techniques of shoulder stabilisation. However, the majority of the published literature, including 2 meta-analyses comparing arthroscopic versus open Bankart repair suggest a poorer outcome in the arthroscopic groups [1,3-6] with recurrence rates of up to 34% being reported. Most of this research is based on older methods of fixation such as staple capsulorrhaphy and transglenoid suturing. More recent developments in technique and instrumentation, most notably suture anchors, are meeting with greater success. There are now several studies showing comparable outcomes between open repair and stabilisation using one of the later arthroscopic methods [7-13]. Knotless anchors arised as a solution to the difficulty of tying secure knots with reliable tension arthroscopically [14]. Bioabsorbable anchors were developed to reduce the risk of metalware infection and chronic foreign body reactions encountered with non-absorbable anchors. Bioknotless anchors are a combination of the above two technologies and consist of Poly(L-lactide) which degrades over 2–3 years, eventually being replaced by the patient's autogenous tissue [15]. The other benefits of arthroscopic surgery, in particular the lack of subscapularis damage, faster return to normal activity, improved range of motion and shorter hospital stay are now being gained without the concomitant increase in recurrence of instability.

## Materials and methods

Between January 2005 and March 2006 15 patients underwent arthroscopic stabilisation of the shoulder using BioKnotless anchors (DePuy Mitek, Raynham, MA). All patients were operated on by a single surgeon (VK) using the technique described below. No patients were excluded from the study and none were lost to follow up. 13 were male and 2 were female. Their average age at time of surgery was 27 (17 to 53). All presented as a result of recurrent traumatic anterior dislocations of which 6 experienced less than 5 dislocations and the remainder more than 5. Each patient was fully assessed by the senior author prior to surgery.

### Operative technique and post-op management

Each patient was examined under anaesthetic prior to arthroscopy. 2 patients had stable shoulders on examination, 11 had anterior instability only and 2 had anterior and inferior instability.

The operative technique has previously been described by Thal et al [14] but for clarity has been included briefly below. Patients were placed in the beach chair position under general anaesthetic plus an interscalene brachial plexus block. Standard posterior and anterosuperior portals were used and arthroscopic evaluation carried out. 10 patients were found to have an isolated Bankart lesion. 4 patients had a Bankart together with a Hill-Sachs lesion and one had a Bankart, Hill-Sachs and a superior labrum anterior to posterior (SLAP) tear.

A 3^rd ^anteroinferior portal is made. The capsule is then mobilised sufficiently to allow repair. The glenoid neck is decorticated using a rasp or burr. An initial anchor hole is made as inferiorly as possible.

**Figure 2:**
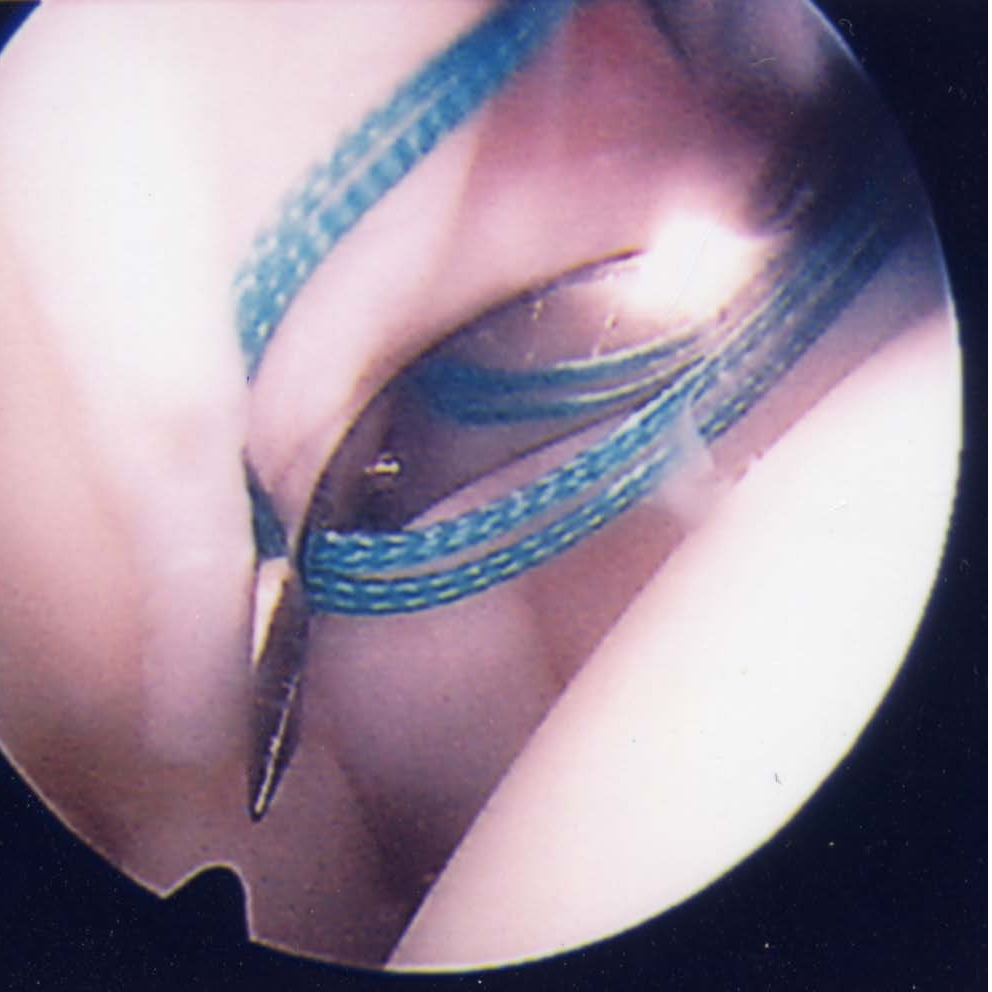
**The shuttle suture (green) is threaded through a sharp device with a slight 'cork-screw' twist**.

**Figure 3:**
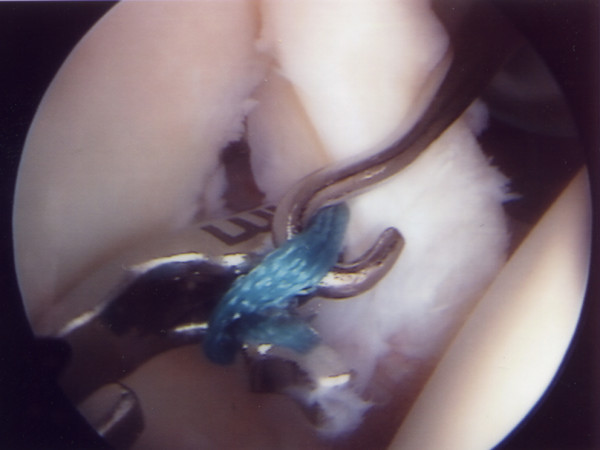
**The shuttle suture is passed through the capsulolabral complex at the desired position**. A hook is used to catch one end of the suture and bring it out the other portal.

**Figure 4:**
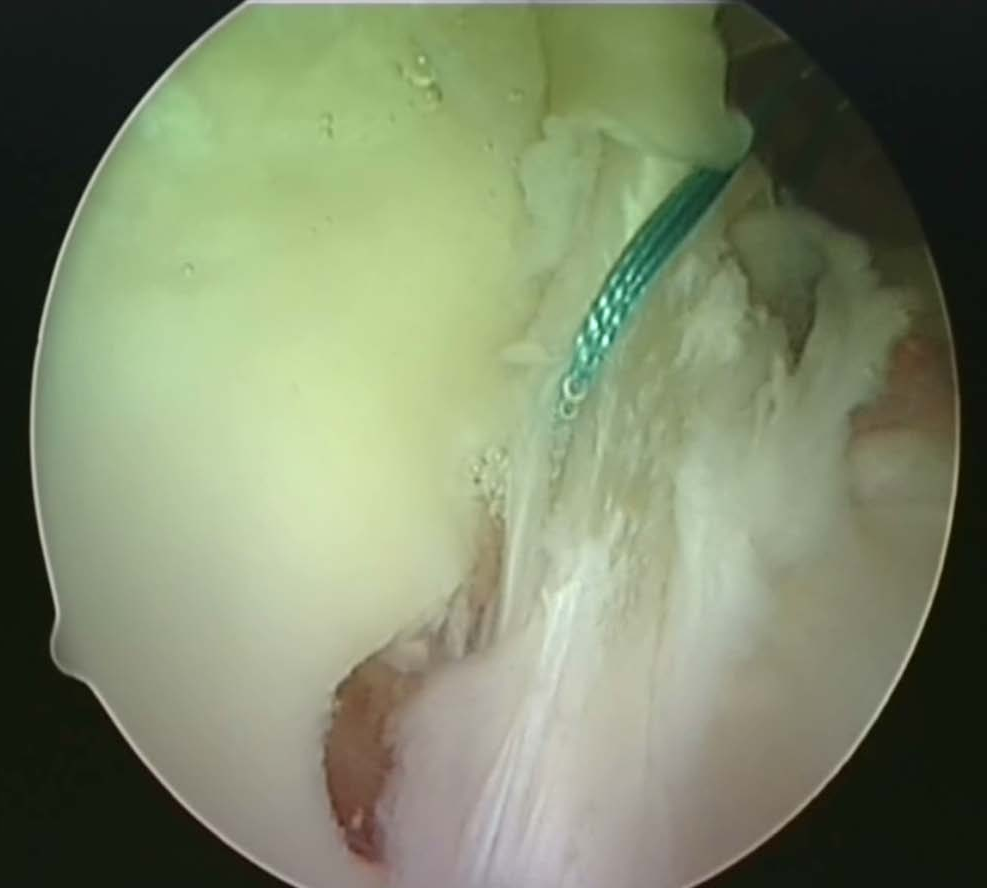
**The shuttle suture (green) is used to pull the anchor suture through the capsulolabral complex**.

**Figure 5:**
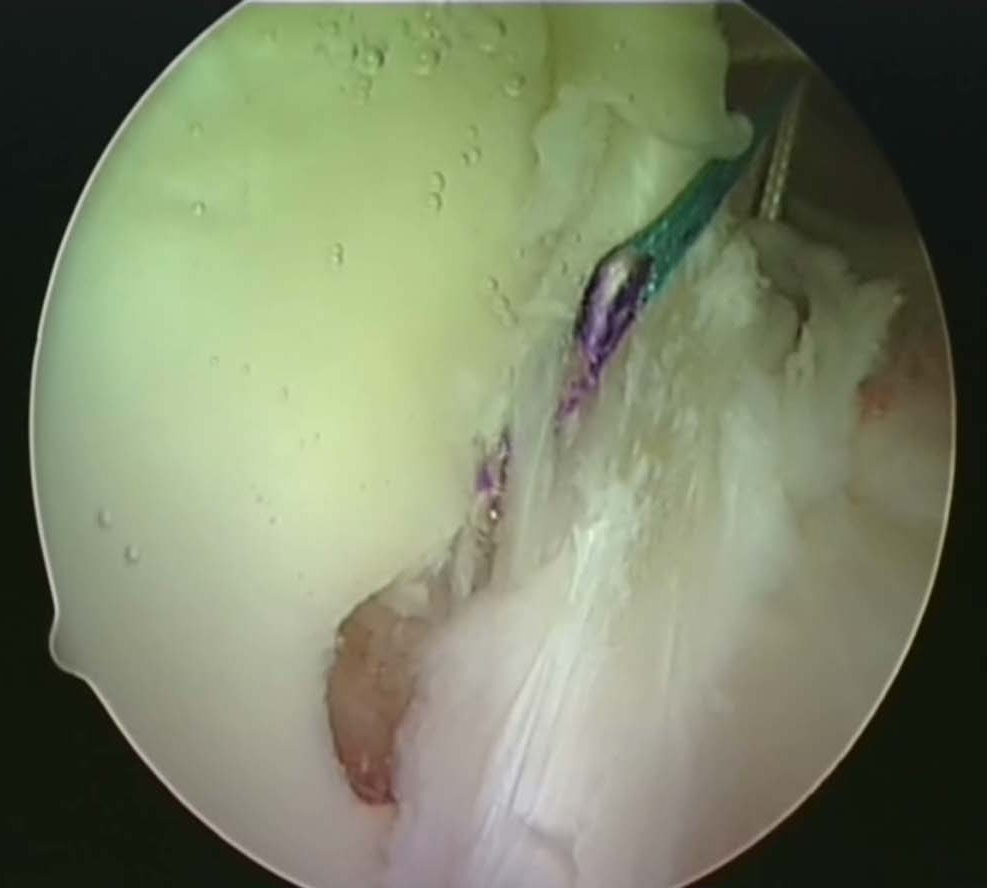
**The anchor suture (blue) is now visible as it passes through**.

**Figure 6:**
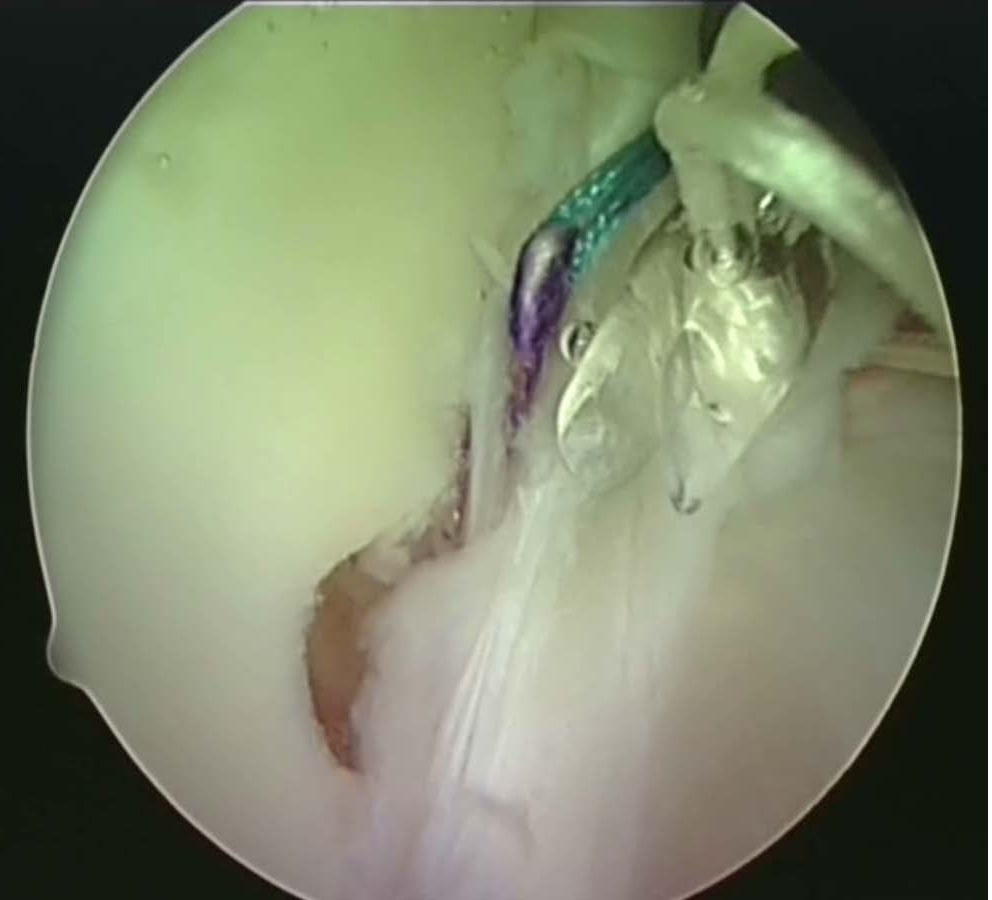
**The BioKnotless anchor is introduced**.

**Figure 7:**
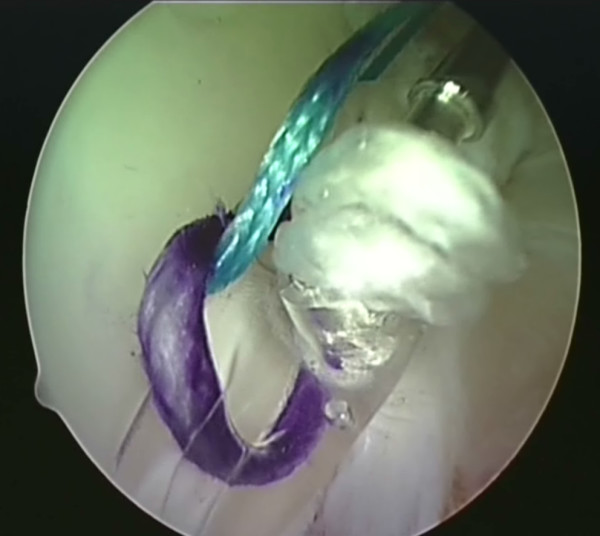
**One thread of the anchor suture (blue) is caught in the teeth of the anchor**.

**Figure 8:**
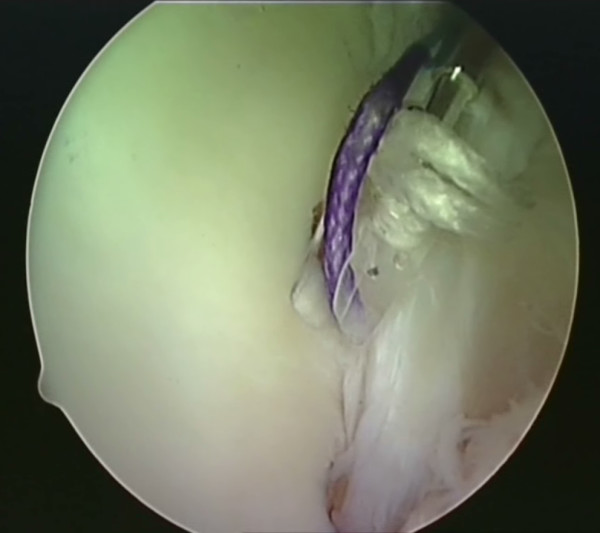
**The anchor plus suture is then lined up with the predrilled hole in the glenoid rim**.

**Figure 9:**
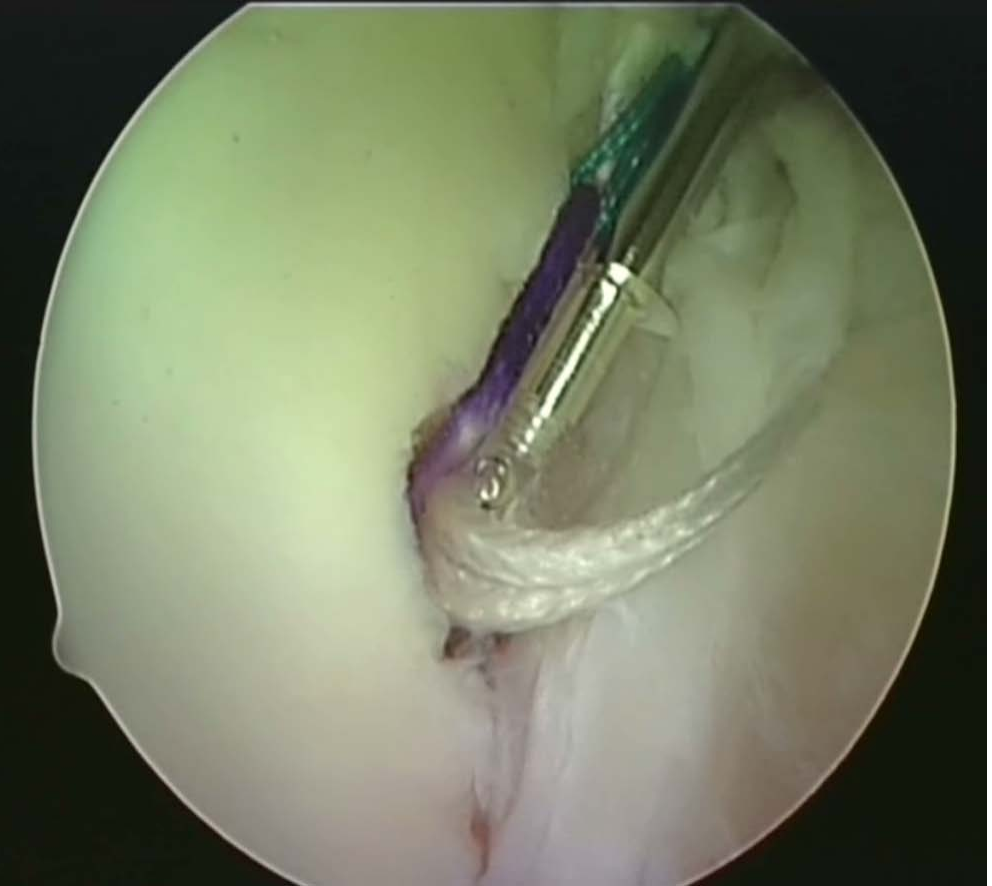
**The BioKnotless anchor is gently tapped into the hole**.

**Figure 10:**
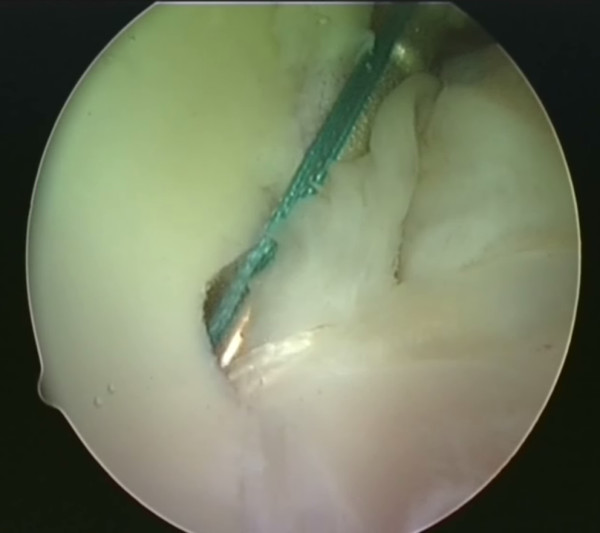
**The anchor is inserted until the desired tension is achieved**. The shuttle suture is then removed.

**Figure 11:**
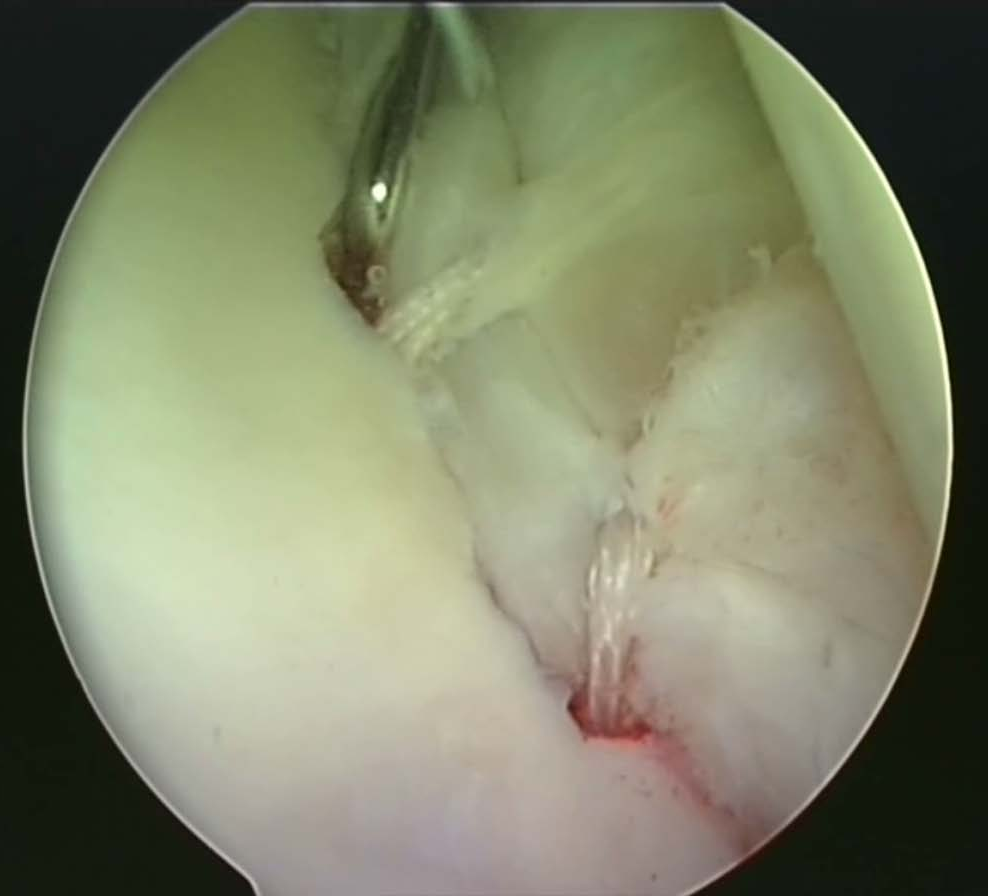
**As many anchors as necessary to effect a complete repair are inserted (two are visible in this view)**. The final repair is probed to ensure good stability.

A utility loop suture is passed through the capsulolabral complex (figures 2 and 3) and used to pull the anchor loop through the soft tissue (figures 4 and 5). The teeth of the anchor are used to capture the loop (figures 6 and 7), which is then inserted into the pre-drilled hole (figure 8). Gently tapping the anchor into the hole brings the soft tissues to the desired position until adequate tension is achieved (figures 9 and 10). This process is then repeated until the entire defect is repaired satisfactorily (figure 11).

All patients are placed in a sling post-operatively. Once pain allows, they are taught pendulum exercises and encouraged to perform these daily. After one week they are reviewed by a physiotherapist who commences passive range of movement exercises. Depending on the individual patient, assisted active and active mobilisation is introduced over the following 6 to 12 weeks. External rotation beyond neutral is avoided for 6 weeks and combined external rotation and abduction for 12 weeks. Contact sports and overhead weight-bearing activities should be avoided for 6 months.

All patients were evaluated post-operatively by the same observer (SJC). Recurrent dislocations, symptomatic instability, patient satisfaction and return to previous activities were recorded. The constant score was measured in both shoulders, the normal side being used as a control. The average length of follow up was 21 months (17 to 31). No patient was lost to follow up.

Ethical approval was received from the audit and research department, Stafford General Hospital, Stafford, UK.

## Results

12 out of 15 (80%) patients were satisfied with their surgery (table 1). 2 patients had recurrent symptoms. 1 had a further dislocation post-operatively (7%) which occurred without major trauma within 6 months of surgery. Following reduction he had no further dislocations or symptoms of instability. Interestingly, this patient was still satisfied with the surgery as at the time of final follow-up, he felt his shoulder was better than prior to the procedure and he had returned to his usual work and recreational activities. A second patient suffered with symptomatic instability and was unable to return to his usual level of sport. Of the 3 patients who were unsatisfied, 1 was because of recurrent symptomatic instability and 2 were due to an inability to return to previous levels of activity. The range of movement measured at final follow up of all 4 patients who were either unsatisfied with the surgery or had recurrent symptoms (or both) is shown in table 2.

**Table 1:** Details of each patient included in the study.

Patient No.	Age	Direction of Instability	Pathology	No. of Anchors	Constant Score (operated limb)	Constant Score (% of control limb)	Post-op Review
1	17	Anterior	B, HS	4	84	88.4	
2	19	Anterior	B	2	50	66.7	SI, U
3	19	Anterior	B	2	82	91.1	
4	21	Anterior	B	3	80	84.2	
5	21	Anterior	B	2	81	84.4	
6	22	Anterior	B	2	86	89.6	
7	23	Anterior	B	2	83	86.5	
8	25	Anterior	B, HS	3	80	84.2	D
9	25	Anterior	B	3	82	86.3	
10	26	Anterior	B	2	73	84.0	U
11	26	Anterior	B, HS, SLAP	4	84	87.5	
12	34	Anterior	B, HS	3	72	78.3	
13	38	Anterior	B	2	76	81.7	
14	38	Anterior	B, HS	3	81	90.0	
15	53	Anterior	B	2	61	69.3	U

B – Bankart lesion, HS – Hill-Sachs lesion, SLAP – superior labrum anterior posterior tear, SI – symptomatic instability, D – dislocation, U – patient unsatisfied.

**Table 2:** Range of motion measured in the patients who were either unsatisfied and/or had recurrent symptoms

Patient No.	Flexion (degrees)	Abduction (degrees)	Internal Rotation	External Rotation
2	61–90	61–90	Lumbo-sacral junction	Behind head, elbow forward
8	121–150	121–150	T12	Behind head, elbow back
10	121–150	151–150	T12	Behind head, elbow back
15	91–120	91–120	Waist	Behind head, elbow forward

The average constant score was 77 ± 9.8 following surgery in the affected shoulder which was 84% ± 7.1% of that measured in the control shoulder. No patient had a score less than 50, 3 had scores between 51 and 75 and 11 had a score of 76 or above.

12 patients returned to their previous levels of work and sporting activity, 5 of whom participated in contact sports. 2 patients who were involved in heavy manual work could not return to their usual job following surgery and 2 patients curtailed their level of sporting activity post operatively.

We experienced no other major complications as a result of this surgery.

## Discussion

The majority of surgeons would agree that recurrent, traumatic anterior dislocation is best treated surgically. The results of conservative management are poor with 75–90% of patients re-dislocating within 18 months of the primary event [16,17]. The timing and technique of surgery is more controversial.

Open repair of the capsulolabral complex has been the most reliable method of avoiding recurrent instability, studies reporting between 3% and 10% risk of further dislocation or instability post-operatively. This compares to recurrence rates as high as 34% following arthroscopic repair [1,4,5,11,18,19]. New developments in arthroscopic surgery along with novel instrumentation, in particular suture anchors, are beginning to redress the balance.

Drawbacks of open surgery are a larger scar, division of subscapularis, slower recovery and rehabilitation and long term reduction in range of movement (particularly forward flexion and external rotation). Although some surgeons believe that it is this reduction in the range of movement that itself improves stability and keeps the shoulder out of the apprehension zone. However, most of the research on suture anchors has only 1 to 3 years follow up, whereas the incidence of recurrent subluxation or dislocation has been shown to increase for up to 7 years post surgery [20]. There are no prospective randomised trials with sufficient length of follow up showing any benefit of arthroscopic over open stabilisation. Furthermore, several trials have reserved arthroscopic repair for the more simple cases and revert to open surgery for the complex patients, thus not comparing like with like. There is also a definite learning curve to be overcome and other complications such as anchor failure [13] and foreign body reactions [12] have been reported.

The return to full activity, including high-risk sports is less predictable than the recurrence of instability. As few as 1/3 of competitive athletes returned to their normal level of sporting activity (especially contact and/or throwing sports) following open repair [18]. 2 of the 3 patients in this study that were dissatisfied with their surgery cited the reason as their inability to return to their former level of activity. Both of these patients suffered no further dislocations or instability post-op and had constant scores of 73 and 61 (84% and 69% of control limb) respectively. One patient played local level cricket and was unable to bowl following surgery. The second had difficulty with heavy lifting and had to change his occupation. Objectively their operations were successful but subjectively they were unhappy. This is probably due to inappropriate expectations of the surgery. Patients should be made aware that there is an excellent chance of curing their instability but a much more variable chance of their returning to normal activities, particularly if they participate in high risk occupations and/or sports.

Some advocate early repair following the first episode whereas other surgeons prefer a trial of conservative management and only operate in recurrent cases. Spatschil et al [21] found that with increasing numbers of dislocations there were significantly higher rates of glenohumeral ligament injuries and Hill-Sachs lesions. Both of these have been found to be associated with a greater risk of recurrence following arthroscopic repair [3] suggesting that early surgery would be beneficial if considering a minimally invasive procedure. In this study, all patients had dislocated at least twice prior to surgery. The mean number of dislocations was 6 (range 2 to 12). One patient was excluded from this calculation as he had had too many dislocations to remember. This patient had recurrent symptomatic instability following surgery. Both patients who had recurrence of symptoms had >5 dislocations prior to surgery.

Boileau et al [3] has identified several risks for recurrence following arthroscopic Bankart repair. Capsular hyperlaxity and a large Hill-Sachs lesion involving >25% of the gleniod articular surface were 2 factors increasing this risk significantly. Out of the 2 patients who experienced recurrence in our study, 1 had a Hill-Sachs lesion which was estimated as involving approximately 20% of the gleniod at arthroscopy. A further risk for recurrence is a bony component to the Bankart lesion. A compression fracture of the anterior glenoid rim greatly increases recurrence rates. Given that the bony anatomy can have such a large affect on outcome, a strong case for pre-operative CT evaluation can be made. One factor which did not appear to be linked with recurrence in our study was the number of anchors used. An average of 2.7 (2 to 4) anchors were used and of the patients with recurrence, 2 and 3 anchors were used respectively. Only 2 patients had 4 anchors placed. The number of anchors used varied on a case by case basis depending on the size of the labral defect to be repaired and the space available on the glenoid rim. It was felt that 2 or 3 bioknotless anchors were ample to affect a sufficiently robust repair of the capsulolabral complex until healing occurs.

## Conclusion

This study shows that the early results of bioknotless suture anchors are in line with similar studies using other suture anchor methods [22,23]. 80% of the patients were satisfied with their surgery and had good or excellent post-operative results with constant scores returning to 84% of that measured in their normal arm. The overall recurrence rate of dislocation was 7% and of instability was 7%. It is further confirmation that new generation arthroscopic methods have similar success rates to open techniques.

## Competing interests

The authors declare that they have no competing interests.

## Authors' contributions

SC helped with the background research, saw all patients at follow-up and wrote the paper with the assistance of the other authors. IS helped with the background research, organised follow-up of patients and assisted with preparation of the manuscript. VK was the senior author, operated on all patients in the study and supervised and advised on the preparation of the manuscript.
